# A review of the design methods of complex topology structures for 3D printing

**DOI:** 10.1186/s42492-018-0004-3

**Published:** 2018-09-05

**Authors:** Jiawei Feng, Jianzhong Fu, Zhiwei Lin, Ce Shang, Bin Li

**Affiliations:** 1 State Key Laboratory of Fluid Power and Mechatronic Systems, College of Mechanical Engineering, Zhejiang University, Hangzhou, 310027 China; 2 Key Laboratory of 3D Printing Process and Equipment of Zhejiang Province, College of Mechanical Engineering, Zhejiang University, Hangzhou, 310027 China

**Keywords:** Complex topology structure, Computer-aided design, 3D printing, Optimization design

## Abstract

As a matter of fact, most natural structures are complex topology structures with intricate holes or irregular surface morphology. These structures can be used as lightweight infill, porous scaffold, energy absorber or micro-reactor. With the rapid advancement of 3D printing, the complex topology structures can now be efficiently and accurately fabricated by stacking layered materials. The novel manufacturing technology and application background put forward new demands and challenges to the current design methodologies of complex topology structures. In this paper, a brief review on the development of recent complex topology structure design methods was provided; meanwhile, the limitations of existing methods and future work are also discussed in the end.

## Background

Complex topology structures, including porous structures, lattice structures, cellular structures, etc. are very common in nature environment [1]. Most of these intricate structures belong to the nonzero genus geometries, which have numerous holes or voids. The complicated topology brings great benefits to the properties.

First of all, with the help of large number of holes, the weight of the geometry model can be significantly reduced; meanwhile, the consumption of energy, material and manufacturing time can also be decreased. Secondly, due to the similarities with natural structures, the porous features of complex topology structures can be applied as human implants or scaffolds for tissue engineering. These porous structures can supply plenty of spaces for cells to attach and proliferate. Thirdly, the complex topology structures can be adopted to achieve multi-functional targets by means of adjusting the parameters of the structures, such as the pore size, pore shape, porosity or specific surface area. The optimum designed structures can be applied for energy absorbing [2], sound attenuating [3], vibration isolating or heat dissipating [4–7].

Although complex topology structures own scores of advantages, they are difficult to be fabricated by the traditional manufacturing processes. Researchers have made several attempts to manufacture complex topology structures by salt leaching, gas-foaming, phase-separation and freeze-drying [8]. Yet, these methods lack enough control of the final pore features. 3D printing, also termed as additive manufacturing, is the state-of-the-art technology to fabricate complex models layer by layer, regardless of the shape geometry [9]. In virtue of the 3D printing technology, complex topology structures can even be manufactured with diverse materials. Nevertheless, the 3D printing technology presents new challenges to the design methodologies of complex topology structures.

Computer-aided design (CAD) technology is an effective tool to design models for 3D printing [10, 11]. The mesh models or parametric models are the two most commonly utilized methods to represent 3D geometries. Yet, they are not ideal for designing all complex topology structures. In order to obtain accurate modeling results, numerous meshes will be utilized to approximate the designed model, which is highly resource-consumptive. Also, dozens of parametric surfaces will be required to merge the whole geometry, which may also bring drawbacks to the final results [12, 13]. With regard to the design task, diverse requirements of design should be taken into consideration, including the mechanical properties, printability and other specific demands. In some cases, the demands of different applications may even be contradictory. For example, Montazerian et al. made a compromise between permeability and elastic properties [14] to design porous scaffolds.

Currently, many researchers have done a lot of work to design complex topology structures for 3D printing according to different requirements. This paper is aimed to supply a brief review of the typical methods of the current design technologies for complex topology structures. The remainder of this paper is organized as follows. In the next section, the design methods for lightweight 3D printing will be briefly reviewed, including several recent novel representative design approaches. As a significant application in tissue engineering, the design methods of porous scaffold will be introduced in Section “Porous scaffold design for tissue engineering”. And in Section “Lattice structure design methods”, the methodology of lattice structure, which is a large branch of complex topology structures will also be discussed. The last section concludes this paper.

## Lightweight structure design for 3D printing

Recently, 3D printing is emerged as an effective technology to efficiently fabricate complex structures layer by layer [11, 15]. Yet, the time- and material-consuming problems are still big challenges of 3D printing. Replacing the solid infill materials with porous structures or other complex topology structures is an effective and convenient solution to the above problems. Note that in order to obtain reliable and lightweight products, the physical properties should also be taken into consideration.

### Special shape structure design

**Fig. 1:**
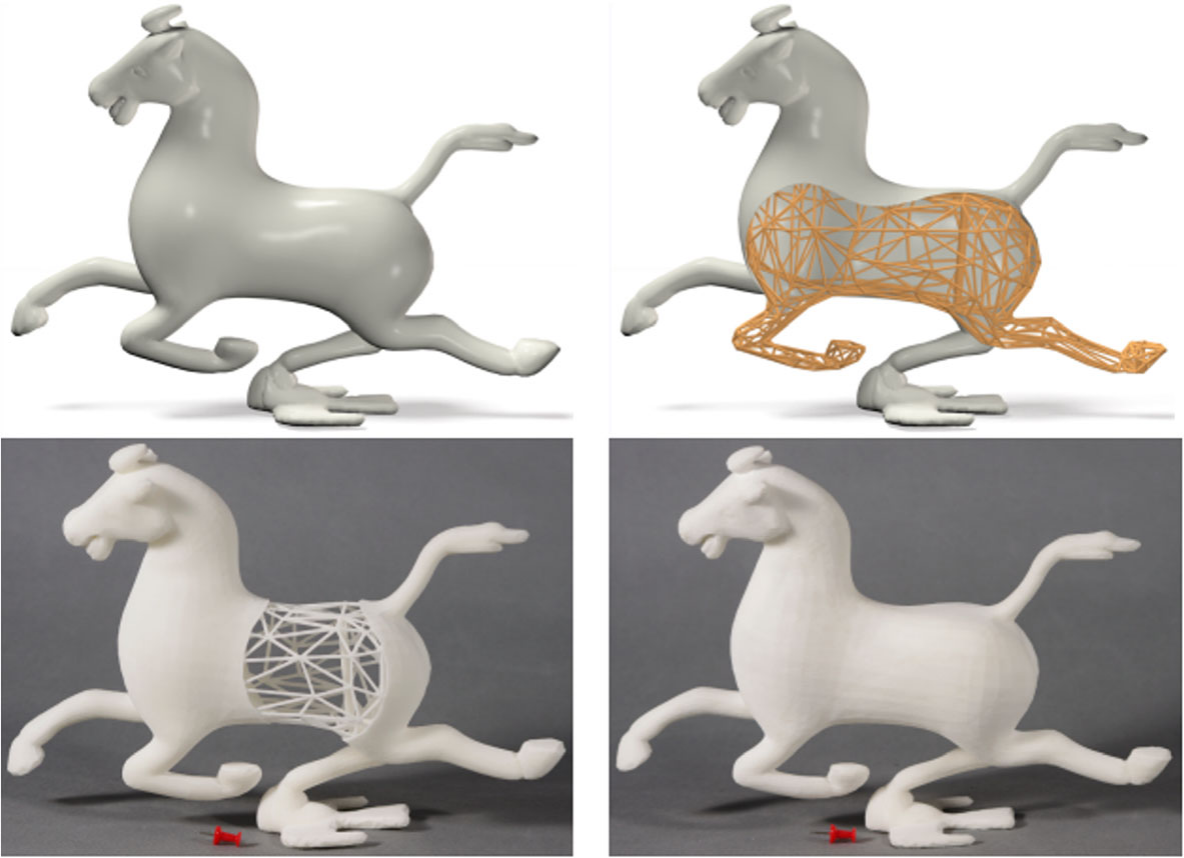
Skin-Frame lightweight structure design and manufacturing results by 3D printing [16]

Inspired by the frame structures widely applied in architecture area, Wang et al. proposed a cost-effective method with skin-frame structures [16]. In their method, the lightweight structure design problem is formulated as a multi-objective programming problem, considering the mechanical properties, balance of models and the printability of corresponding technology. After numerous iterative calculations, a compromise solution can be acquired to generate feasible lightweight models, as presented in Fig. 1.

**Fig. 2:**
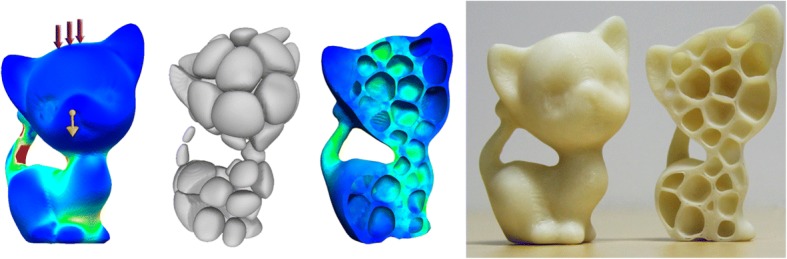
Honeycomb-like lightweight structure design [17]

Hollowing is another convenient and direct way to reduce the material consuming. The solid model can be hollowed into a thin wall with constant thickness. With the significant decrease of weight, the mechanical strength of the generated printing model is difficult to guarantee. Lu et al. introduced a lightweight scheme based on the honeycomb-cell structures [17]. The Voronoi diagram was utilized to compute irregular honeycomb-like volume tessellations. The strength-to-weight ratio can be effectively optimized by their method. Nevertheless, as shown in Fig. 2, the generated pores of this method is unconnected, which may cause troubles to the powder sintering-based manufacturing technology.

**Fig. 3:**
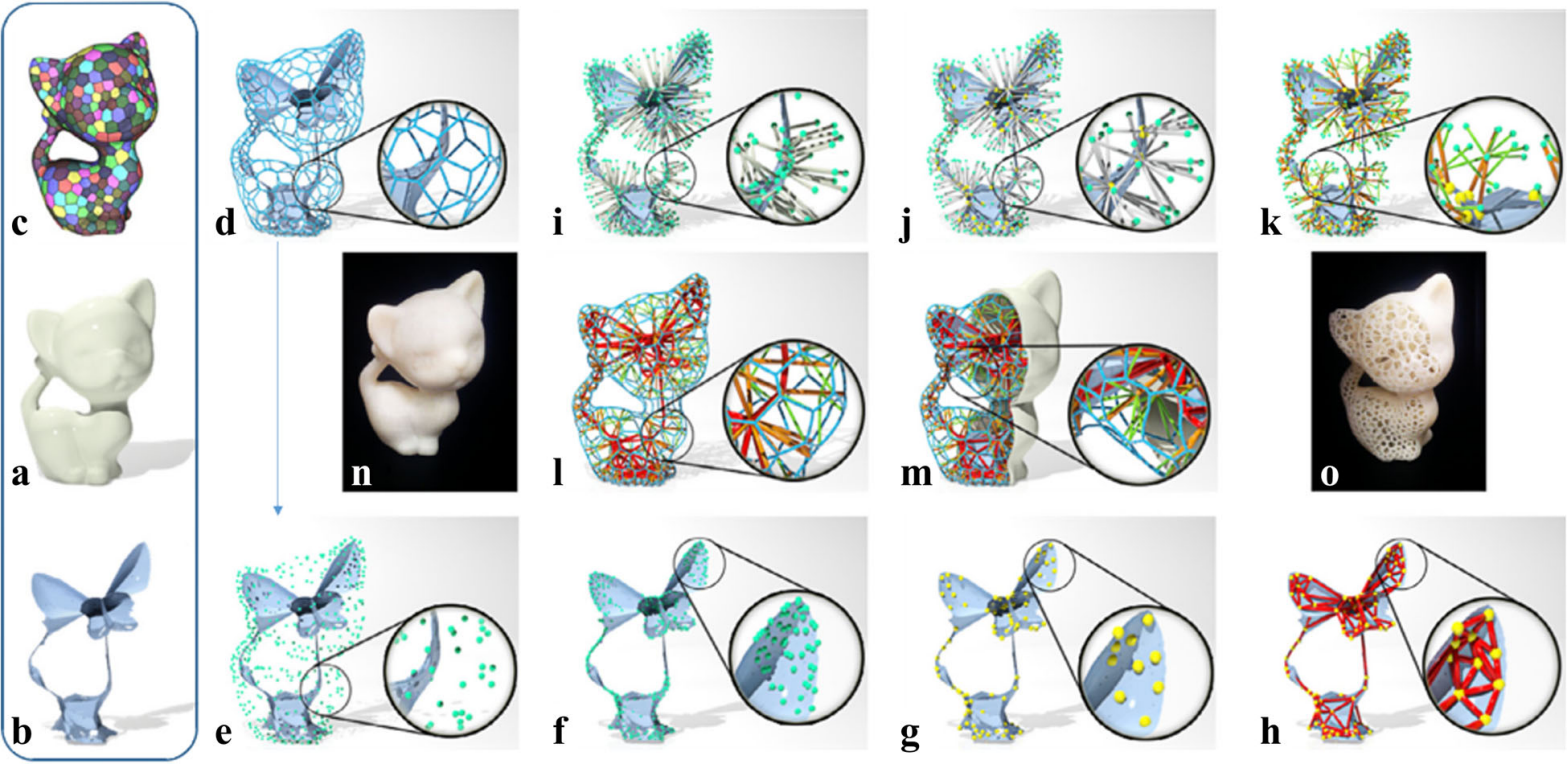
Lightweight structure design method based on the media axis algorithm. **a** Origin model. **b** The media axis. **c** Restricted centroid voronoi tessellation. **d** Hexagonal frame. **e**. Sampling points. **f** Projection in media axis. **g** Group nearby points. **h** Resulting frame. **i** Connecting results of projection point. **j** Connecting results of group points. **k** Adaptive connecting results. **l** Details of the final structure. **m** Final frame. **n** Addictive manufacturing of origin model. **o** Addictive manufacturing of the lightweight structure [18]

The art of the lightweight structure design for 3D printing is to find a balance between the material-consuming and physical performances. The media axis algorithm was adopted to design lightweight complex structures [18]. The hexagon framework approximate the original boundary surface of the model. Meanwhile, branching bars are utilized to connect the framework with these generated media axes, as shown in Fig. 3. Thus, the designed structures can withstand loads from multi directions, which is more commonly existed in actual application situations.

**Fig. 4:**
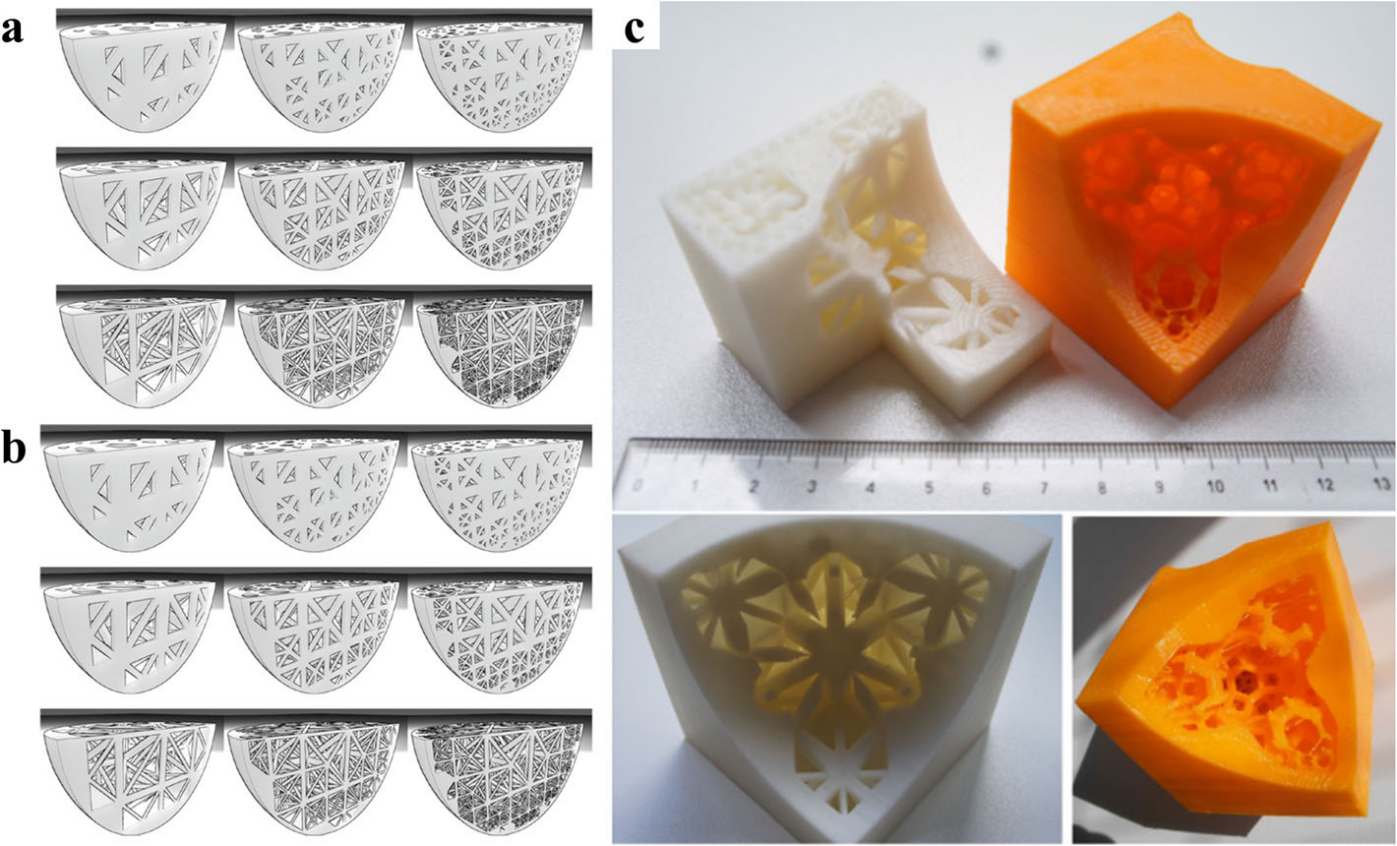
Lightweighting structures: **a** Primal cellular structures. **b** Dual cellular structures. **c** Manufacturing results [19]

In order to reduce material consumptions while maximizing the mechanical strength, Sá et al. presented an adaptive voids algorithm to hollow the 3D printing models [19], as shown in Fig. 4. This method is feasible to generate configurable voids with specific density by the parametric algorithm framework, which can be conveniently applied in the actual application-specific situations. Compared with other design methods, this approach offers merits in the automation and configurability.

**Fig. 5:**
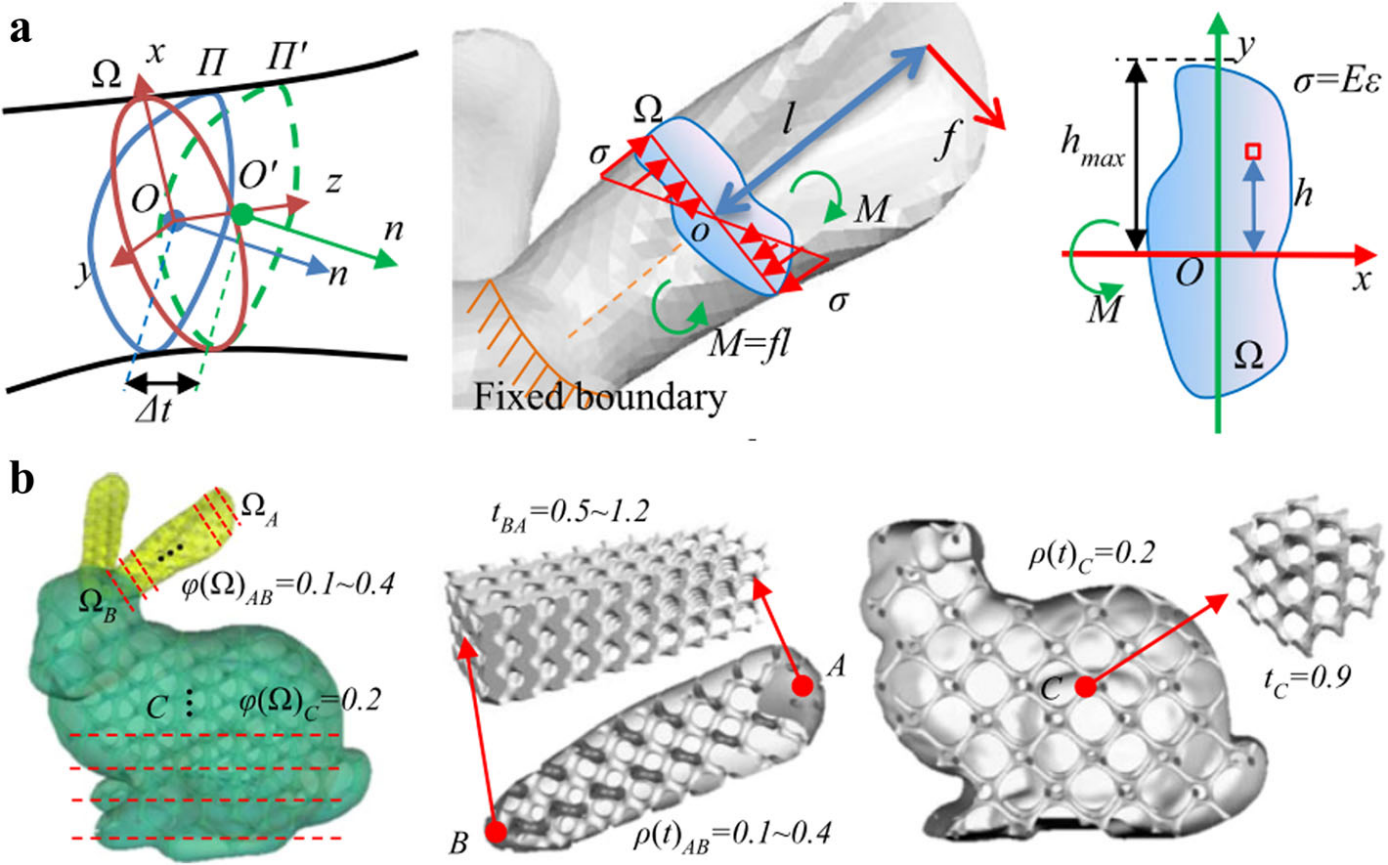
Density-variable Lightweight structure design: **a** Cross section analysis. **b** Infill porous structures with Gyroid structures [20]

In most cases, the mechanical analysis is the crucial task of lightweight complex structure design for 3D printing. With the help of the cross section analysis method, Li et al. proposed a novel density-variable lightweight structure design solution [20]. In virtue of changing the density of Gyroid structures, the designed results can fully satisfy the mechanical requirements. More importantly, the generated pores are highly interconnected as presented in Fig. 5, which is beneficial for powder sintering-based 3D printing.

**Fig. 6:**
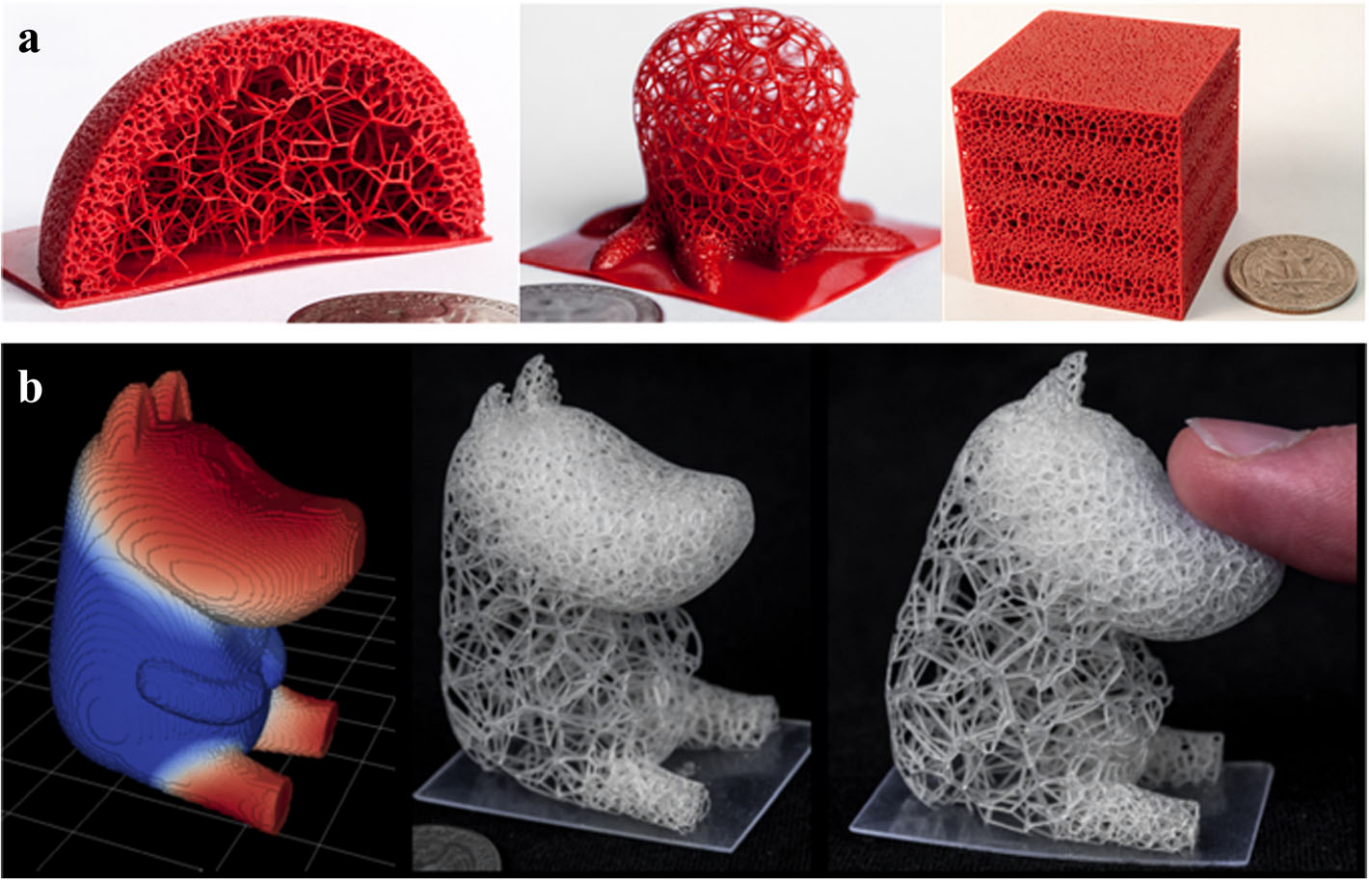
Voronoi foam design for additive manufacturing: **a** The design and fabrication results. **b** Graded material elasticities for load bearing [21]

Moreover, foam structure is also a common lightweight architecture widespread in nature. Considering the intricate structures with complex topology, a precise and convenient approach to describe the geometry shape and physical performances is a challenge in CAD domain. Martínez et al. made use of the Voronoi foams to design and generate microstructures with controlled isotropic elastic behavior [21], as shown in Fig. 6. According to the required elasticity, the ideal parameter can be efficiently obtained to construct corresponding structures. With the help of the proposed implicit formulation of the designed microstructures, many computational resources can be saved by avoiding the conventional complete representations, such as the mesh or voxel methods.

**Fig. 7:**
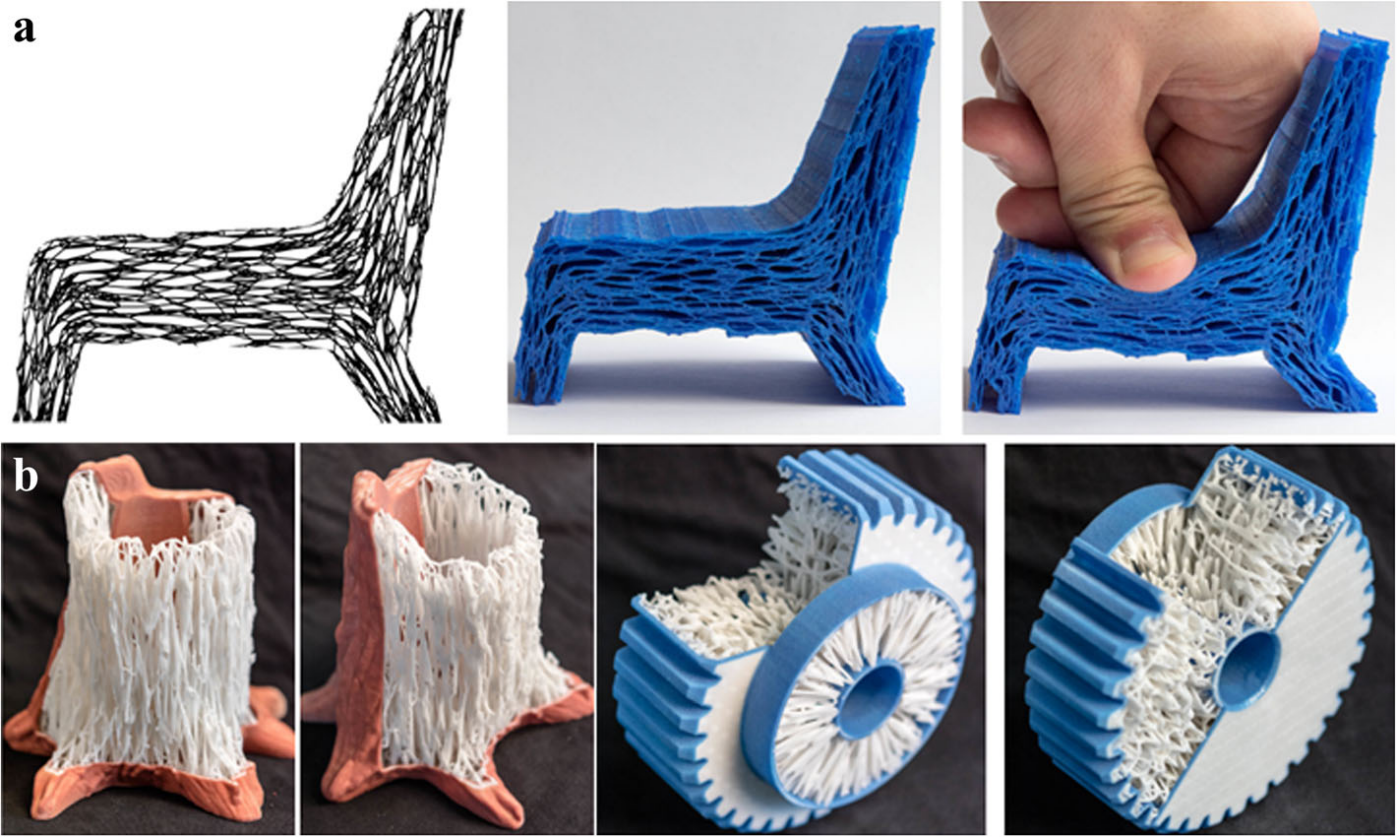
Orthotropic k-nearest foam design: **a** Orthotropic design for loads from different directions. **b** Fabrication models with orthotropic filling structures

For the purpose of further improving the degree of design freedom of complex topology structures, Martínez et al. presented an orthotropic k-nearest foam design method for additive manufacturing [22]. The elasticity of the constructed structures can be independently controlled along different directions. Hence, the ideal porous foams can be designed and fabricated according to the input stress field, as presented in Fig. 7.

**Fig. 8:**
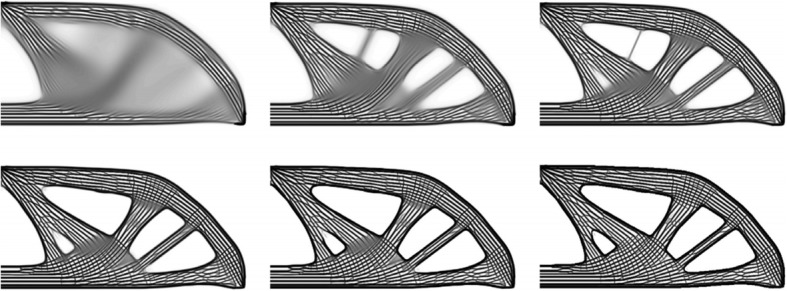
The iteration process of topology optimization [23]

The topology optimization is an important design method, which attracts more and more research attentions. The shell-infill composites are widely applied in engineering, especially on the current additive manufacturing technology. Wu et al. presented a topology optimization method for the shell-infill composites [23]. According to the principle stress directions, the porous infills and the solid shell are optimized at the same time. The iteration process of the density distribution is illustrated in Fig. 8.

**Fig. 9:**
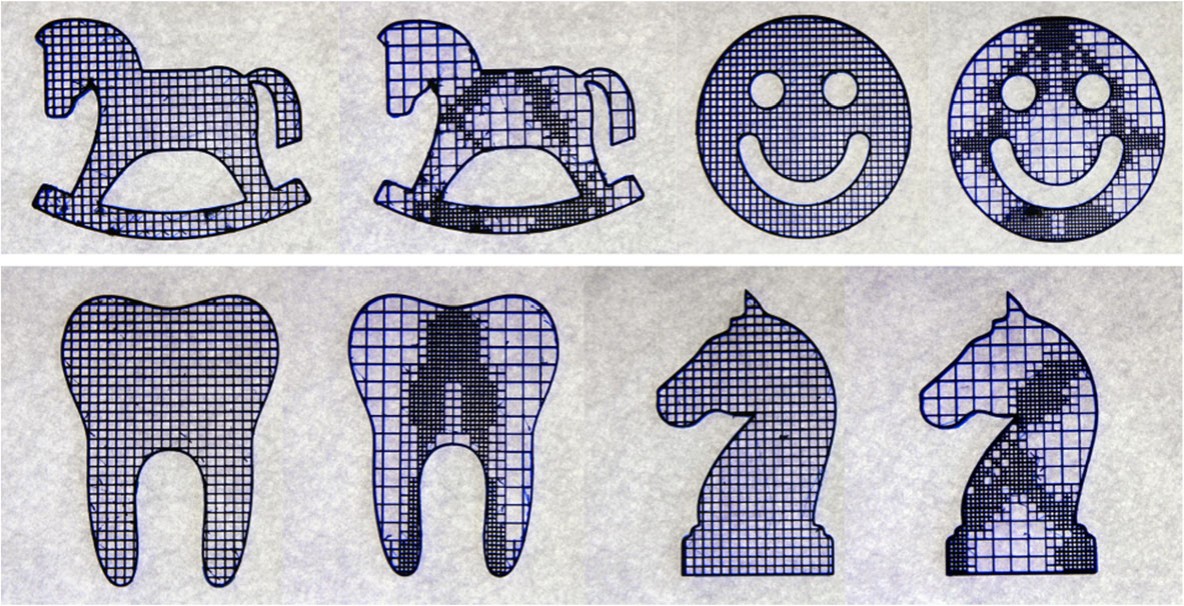
The 3D printing results after topology optimization [24]

The infill structures have significant influences on the performances of products manufactured by AM. Denser infill materials will supply stronger support, meanwhile costing more fabrication time. Wu developed a novel continuous optimization approach based on the quadtree [24]. As presented in Fig. 9, the optimized infill structures can meet the required performances with less material than the conventional pattern.

### Special data structure design

**Fig. 10:**
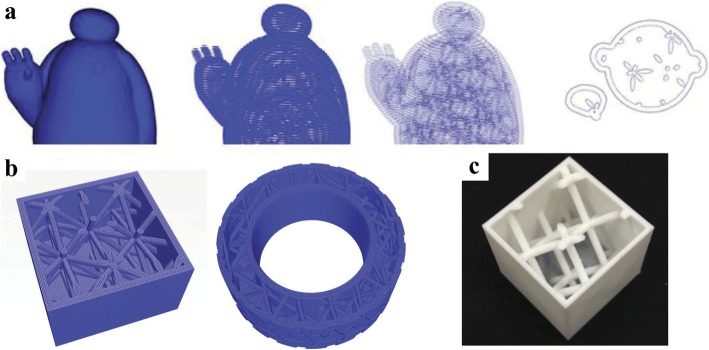
Lightweight structure design based on LDNIs: **a** Generated infill paths for 3D printing lightweight structures. **b** Infill structures. **c** Manufacturing result [25]

All the above generation methods of porous lightweight structures, including truss or honeycomb structures require a great deal of computing resources. In order to precisely generate the truss structures inside models as infill support, Zhao et al. developed an efficient design method based on implicit representation [25]. The designed truss structures are constructed via layer-depth normal images (LDNIs), which can significantly simplify the three-dimension Boolean operations into only one-dimension [26, 27]. The original model can be efficiently hollowed, then combined with the designed truss structures, which is illustrated in Fig. 10.

Chougrani et al. also noticed the oversize problem of lightweight structures [28]. They proposed a special data structure to construct less triangles than typical methods with full consideration of the chord error. And this approach can also be expanded to design non-uniform lightweight structures. Yet, they only supplied the design method of beam unit, which is only one case of lightweight structures.

So far, lightweight design is not only a significant task, but also an attractive topic for 3D printing. Currently, many researchers have made plenty of attempts to design lightweight products, considering the mechanical requirements. Yet, the design and optimization methods in more complicated situations, such as the products applied in multi-physical environment, deserves more research attentions. More universal and convenient algorithms for lightweight structure design are remain to be developed.

## Porous scaffold design for tissue engineering

As an essential support for cell multiplication in tissue engineering, porous scaffold is a typical kind of complex structure, requiring reasonable design with full consideration of multidisciplinary demands [8]. In order to supply enough space for cell proliferation or nutrition transportation, the ideal porous scaffold should be high-porosity with interconnected and non-tortuous pores. With the aid of 3D printing, this kind of complex structures can be directly manufactured layer by layer. Yet, the design of porous scaffolds for 3D printing is still a challenge [29].

### Scaffold design with TPMS

**Fig. 11:**
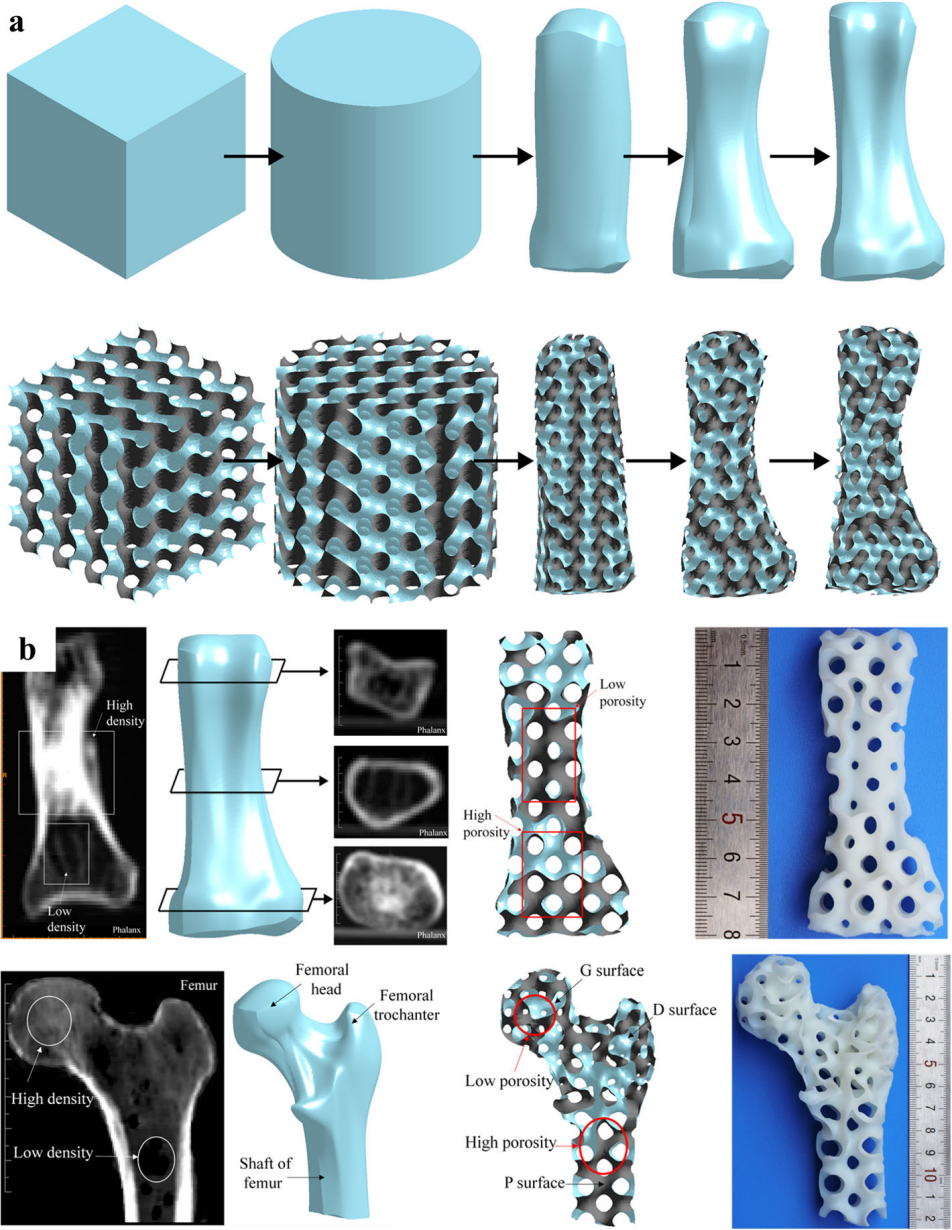
Porous scaffold design by solid T-splines and TPMS: **a** External shape design. **b** Internal structure design [31]

The triply periodic minimal surface (TPMS) is a kind of implicit surface with intricate structures, which has captured great research interests all over the world [30]. In order to conveniently design both the external shape and internal architecture of scaffolds, researchers have made many attempts. Feng et al. proposed a novel design method for scaffolds with the help of solid T-splines and TPMS [31]. The external shape of the scaffold is adjusted by modifying external control points of solid T-splines; meanwhile, the internal complex porous structures are designed by these internal control points according to the actual porosity distribution acquired from CT images, as presented in Fig. 11. The salient feature of their method is that the scaffold can be locally modified without changing the overall shape in virtue of the local refinement algorithm inherited from T-splines.

**Fig. 12:**
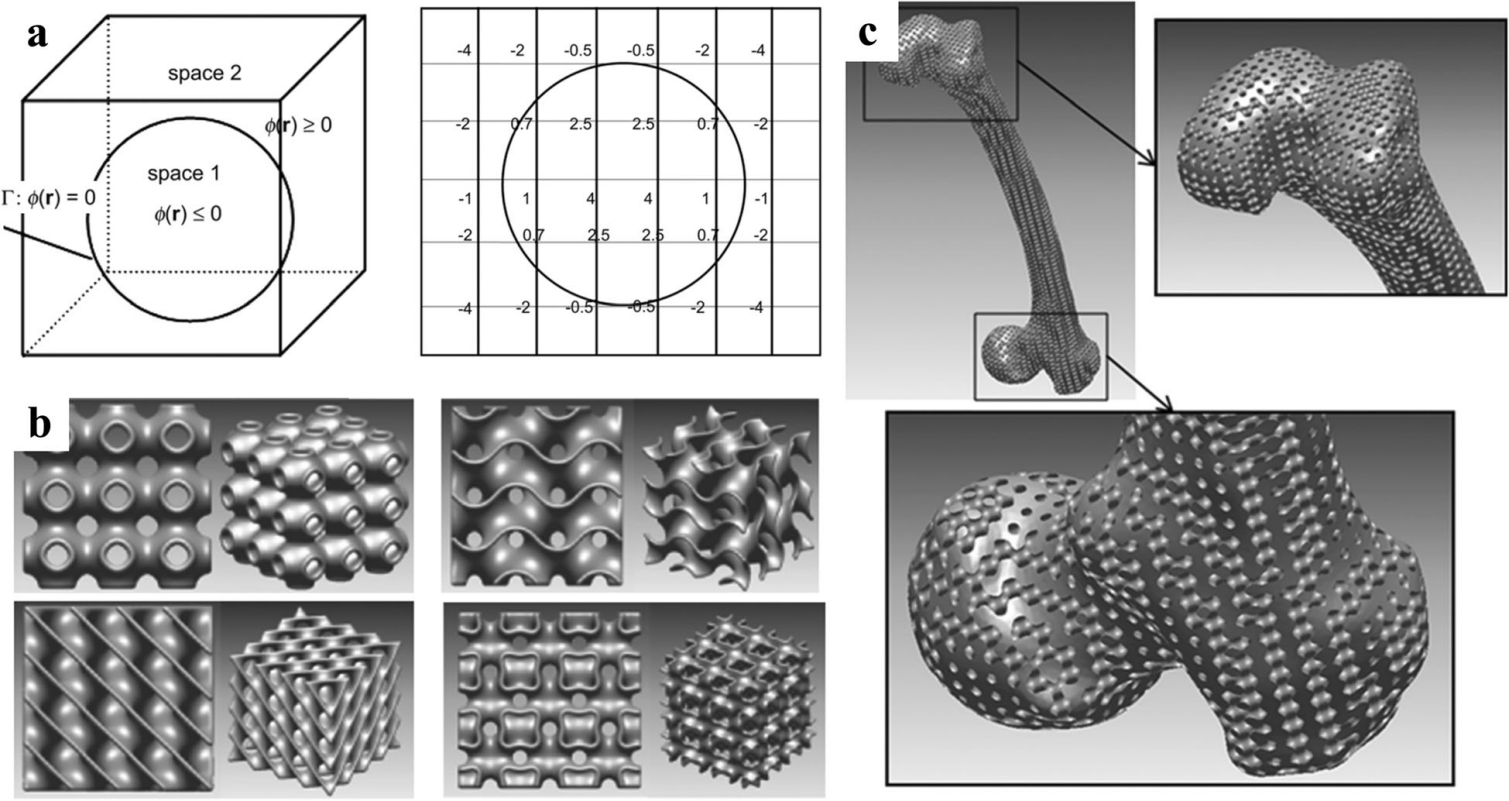
Porous scaffold design by the distance field and TPMS: **a** Schematic diagram of the distance field. **b** TPMS units. **c** Femur bone scaffold composed of P-surface internal architecture [32]

In order to efficiently combine the intricate TPMS with the designed external shape, Yoo presented a hybrid method of distance field and TPMS to design porous scaffolds [32]. After applying the Boolean operations of the anatomical model and TPMS-based unit cell libraries, defect-free porous scaffolds with complicated micro-structures can be generated as shown in Fig. 12.

**Fig. 13:**
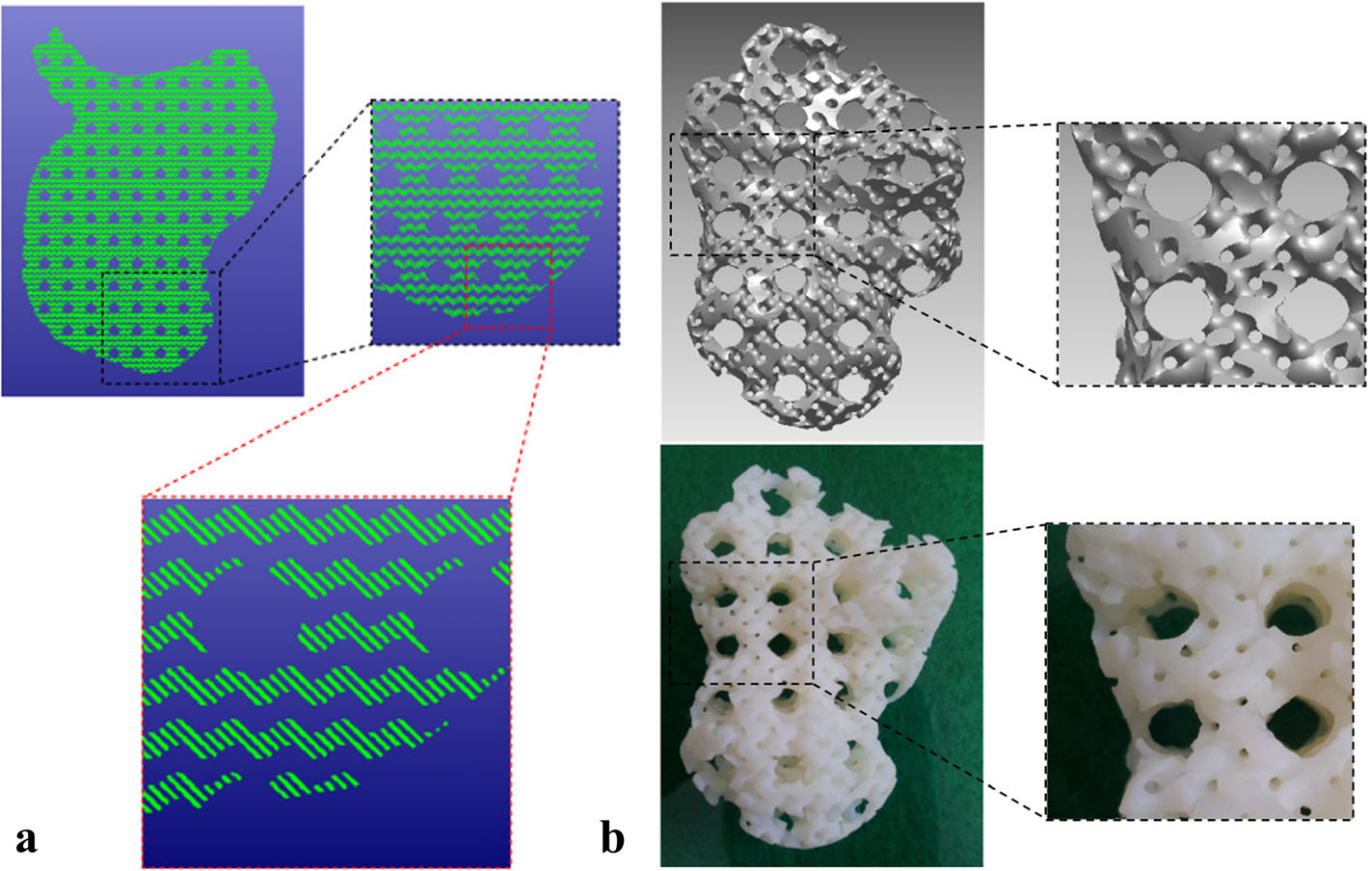
Hierarchical TPMS scaffold design: **a** Macrostructure, mesostructure and microstructure. **b** Manufacturing result by 3D printing [33]

Similarly, the generated TPMS scaffolds can be intersected with TPMS libraries again by the distance field to generate hierarchical TPMS scaffolds [33], as shown in Fig. 13. Thus, by means of the Boolean operations with TPMS libraries in different scales, the complex pores can be designed with multi-scale complex topology structures, including macrostructures, mesostructures and microstructures, which can supply varies functions in scaffolds. Note that the Boolean operations are time-consuming and error-prone, especially when the scaffold models are designed with numerous triangle facets. This process may even be repeated several times to obtain ideal results.

**Fig. 14:**
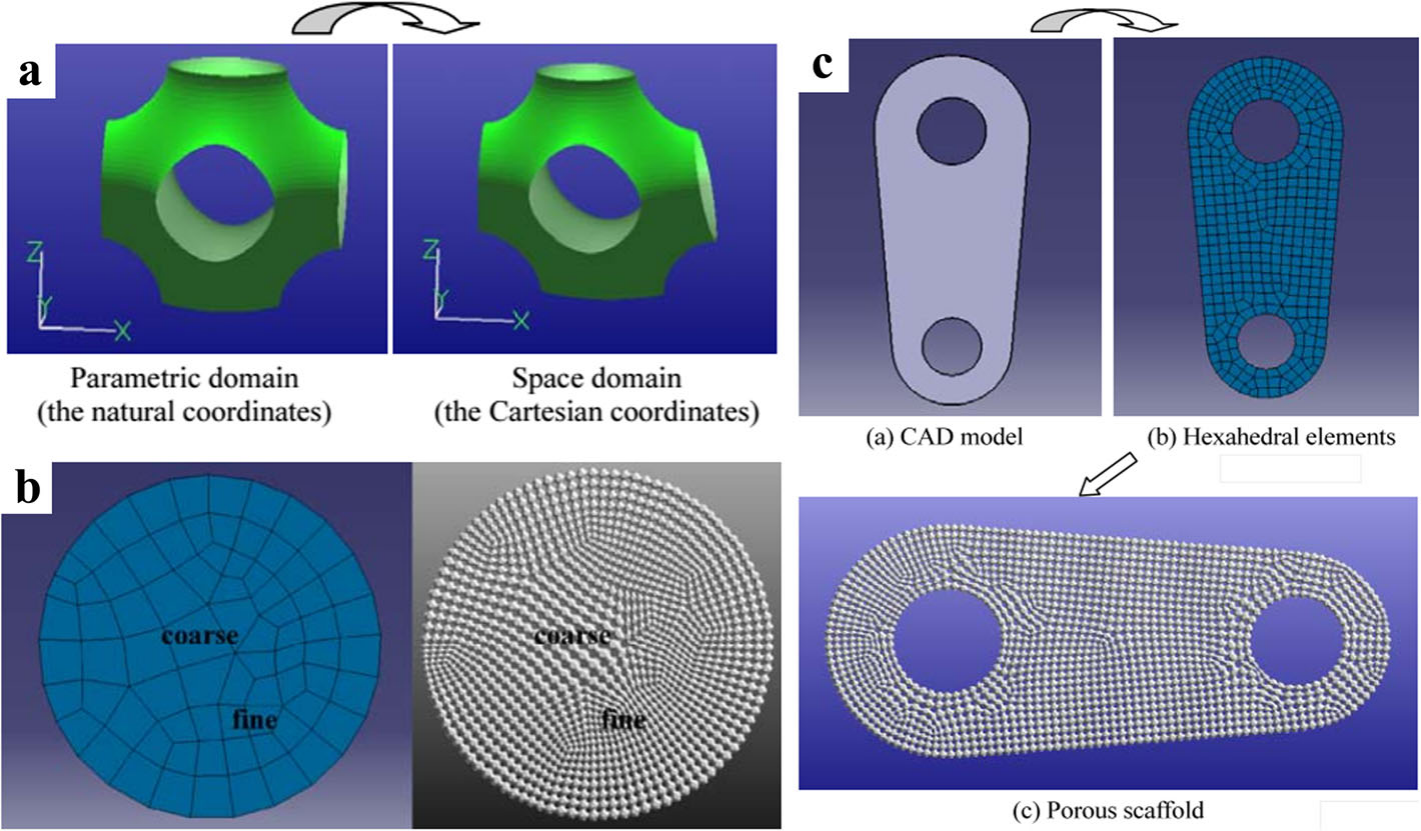
Porous scaffold design by the shape function and TPMS: **a** The mapping method. **b** Mesh division. **c** The generated TPMS scaffold [34]

Inspired by the meshing method of Finite element method, Yoo further made use of the shape function to map the TPMS units from parametric domain to the space domain [34], as shown in Fig. 14. Thus, the pore size distribution can be controlled by changing the density of hexahedral elements. On one hand, the porosity can be locally modified by this method; on the other hand, the mapping process may distort the surfaces of TPMS, which lose the smooth advantages of minimal surfaces and may even obstruct cells to attach on the scaffolds.

### Scaffold design by graphic methods

**Fig. 15:**
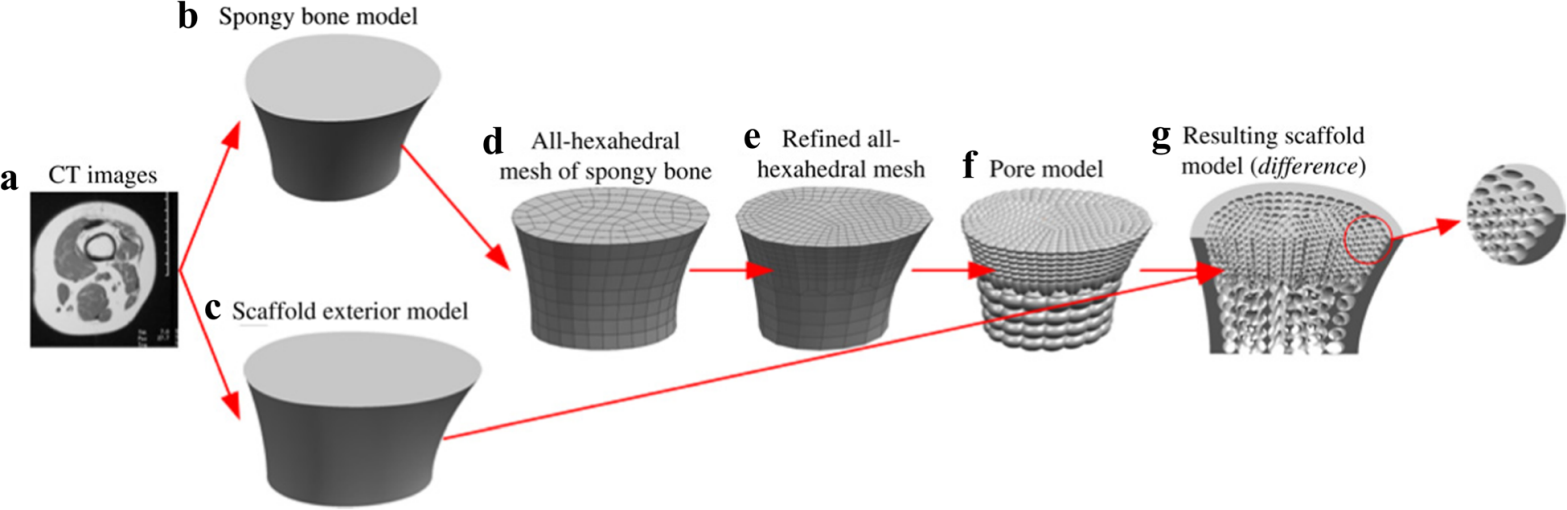
Scaffold design method based on shape function and sphere pores [35]

In order to accurately design the internal architecture of complex topology structures, plenty of graphic methods were utilized. Cai and Xi also made use of the shape function to design porous scaffolds, as illustrated in Fig. 15. However, the sphere is selected as the basic pore-making element for mapping [35]. Apparently, the pore size distribution can be controlled by the hexahedral mesh refinement algorithm. Yet, the generated corners after the Boolean operations between elements and spheres are sharp, which may be harmful for the cell growing.

**Fig. 16:**
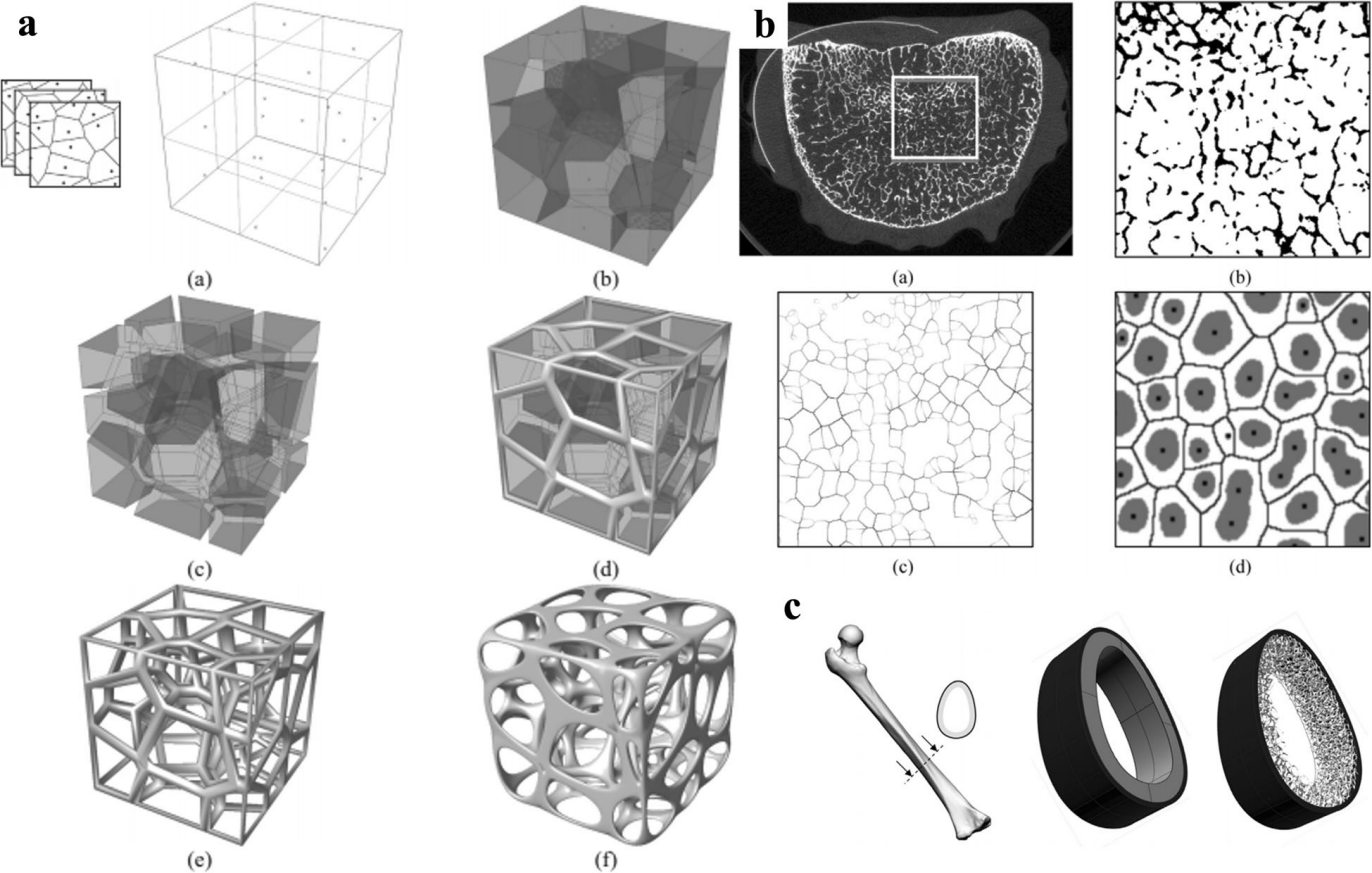
Design method of scaffold: **a** 3D Voronoi to generate porous structures. **b** Voronoi tessellation from CT images. **c** Generate infill scaffolds as bone implant [36]

**Fig. 17:**
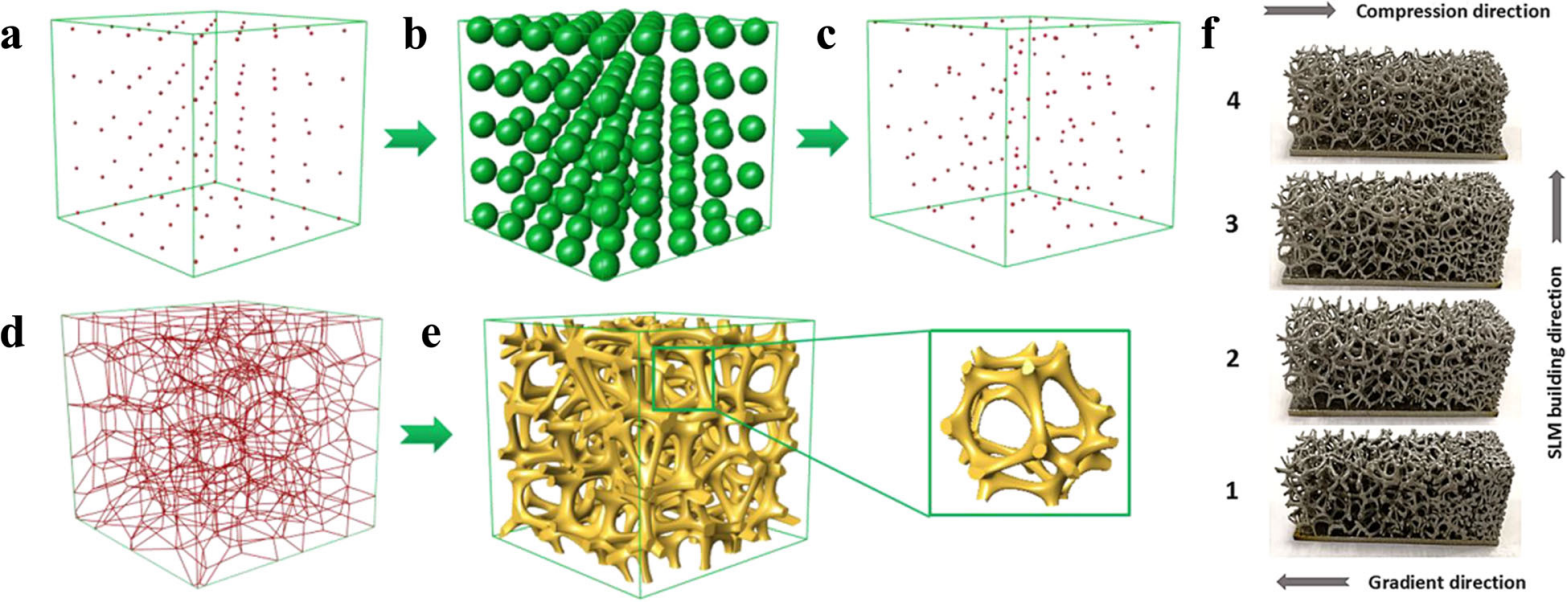
Design process of porous scaffold based on Voronoi-Tessellation. **a** Lattice generation. **b** Sphere construction. **c** Irregular result. **d** Voronoi frame generation. **e** Resulting porous scaffold. **f** Addictive manufacturing [39]

**Fig. 18:**
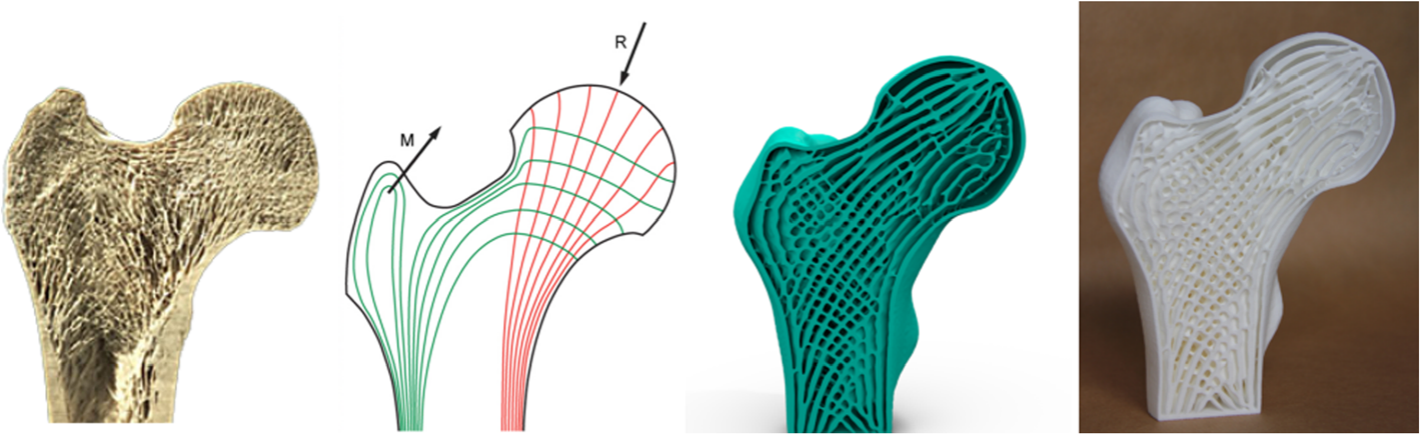
Infill design on account of the principal stress directions and the 3D printed bone model [40]

**Fig. 19:**
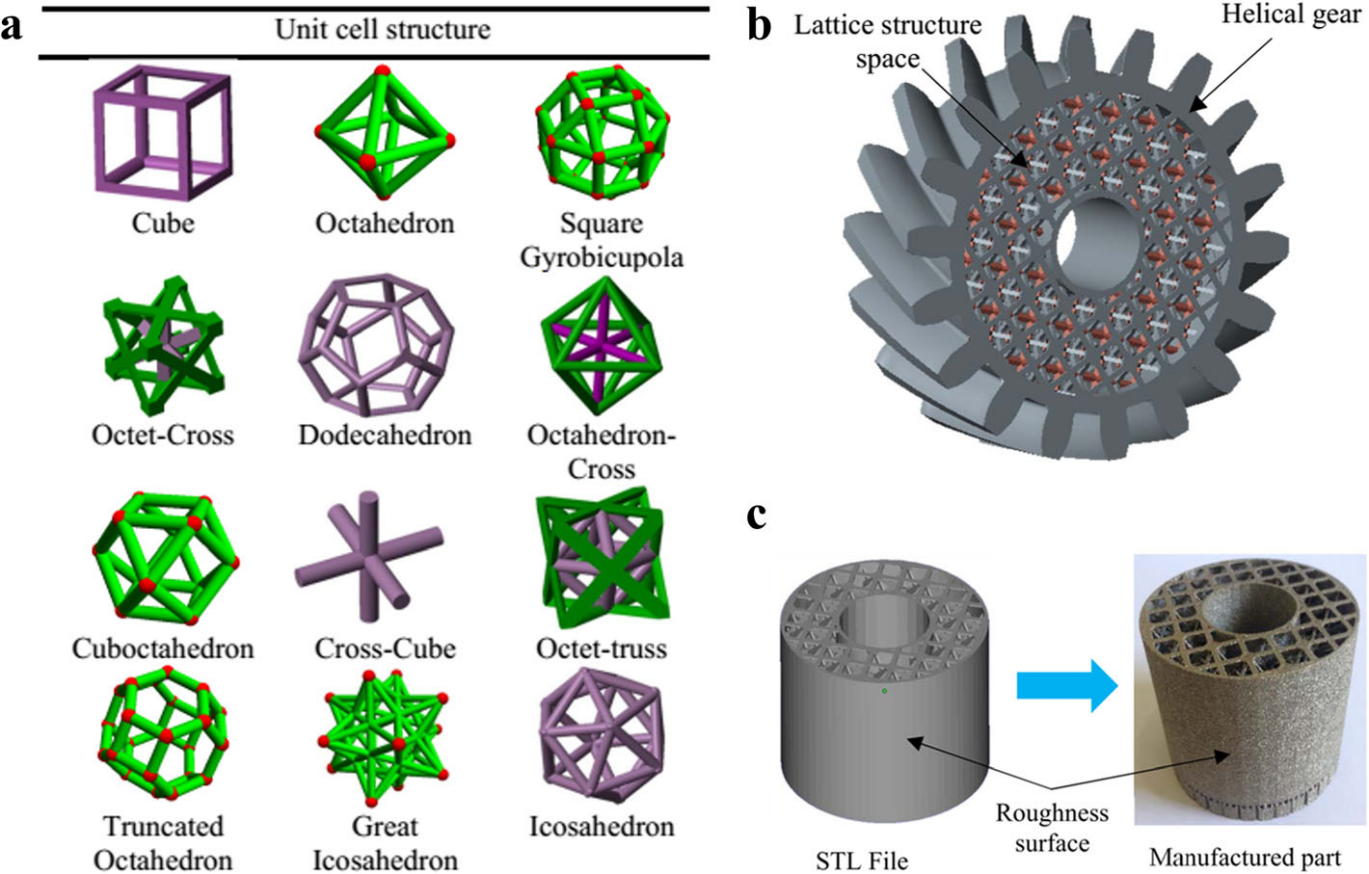
Lattice structure design based on the unit libraries: **a** Unit cells. **b** Designed products with lattice structures. **c** Manufacturing result by 3D printing [6]

**Fig. 20:**
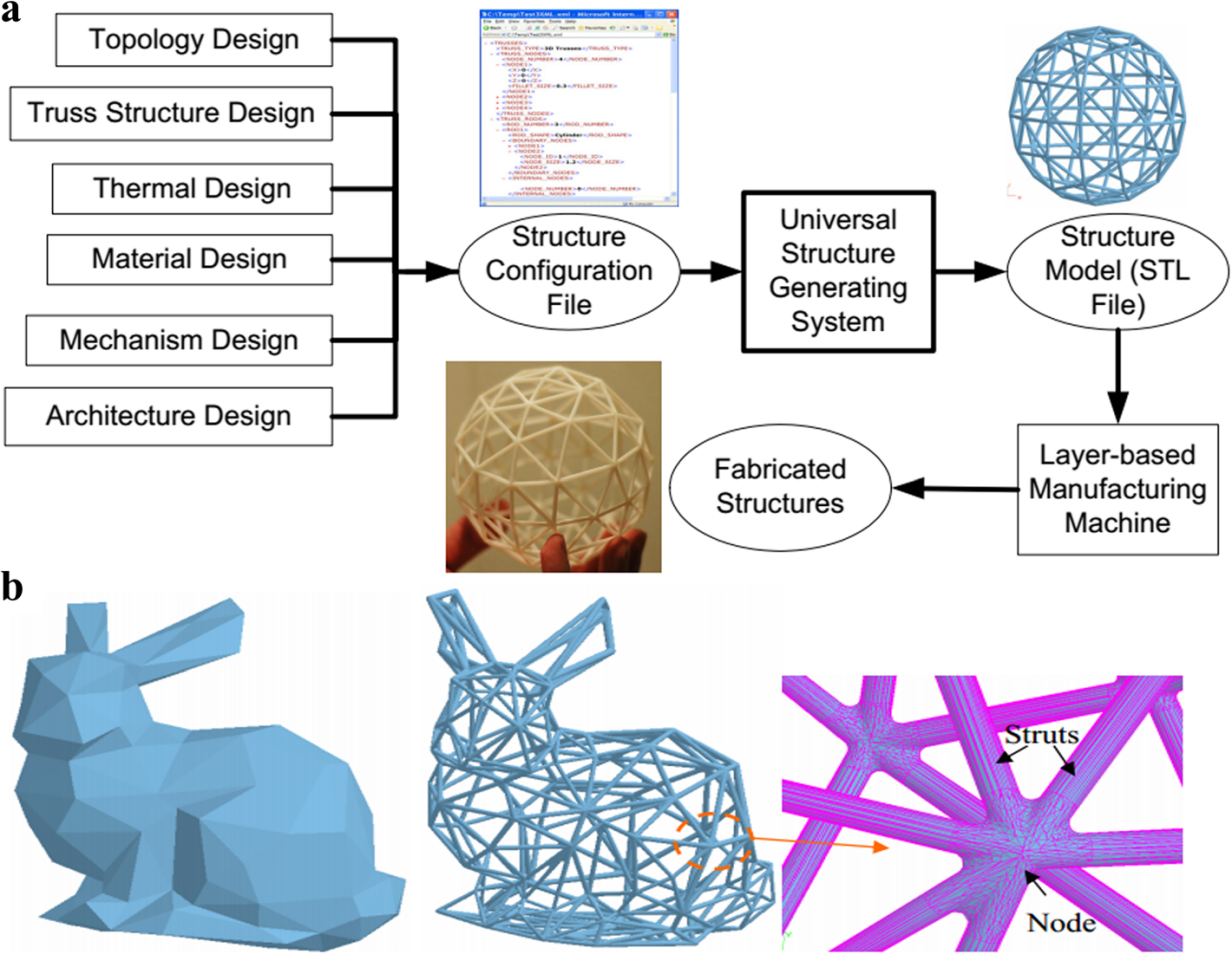
Lattice design system: **a** The universal design system. **b** Design results of lattice structures [12]

**Fig. 21:**
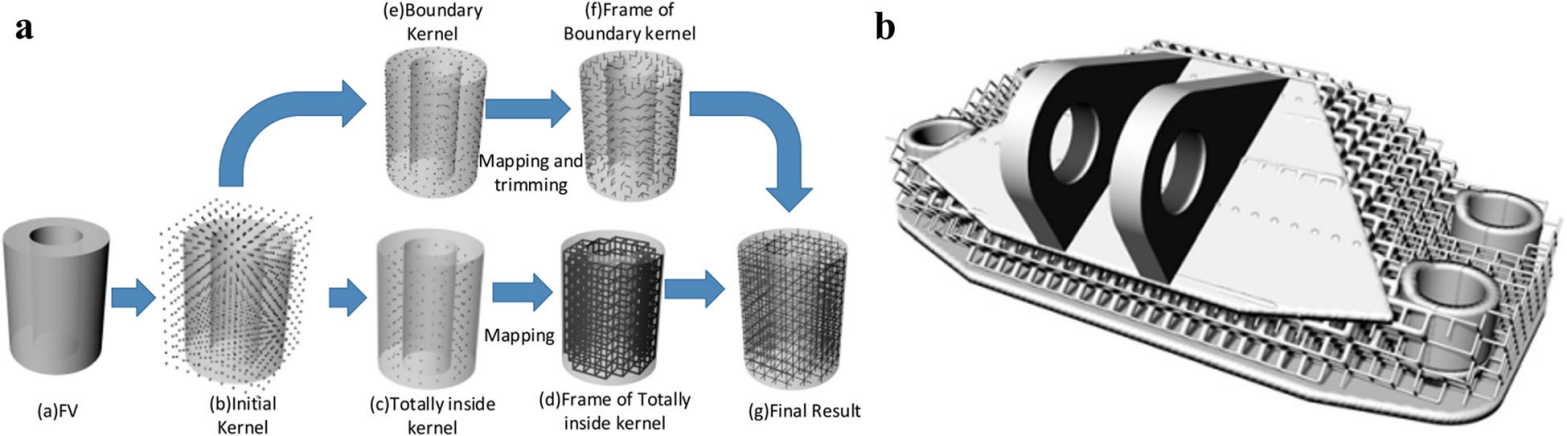
BESO-based lattice design method: **a** The whole design process. **b** Optimized lattice result [51]

**Fig. 22:**
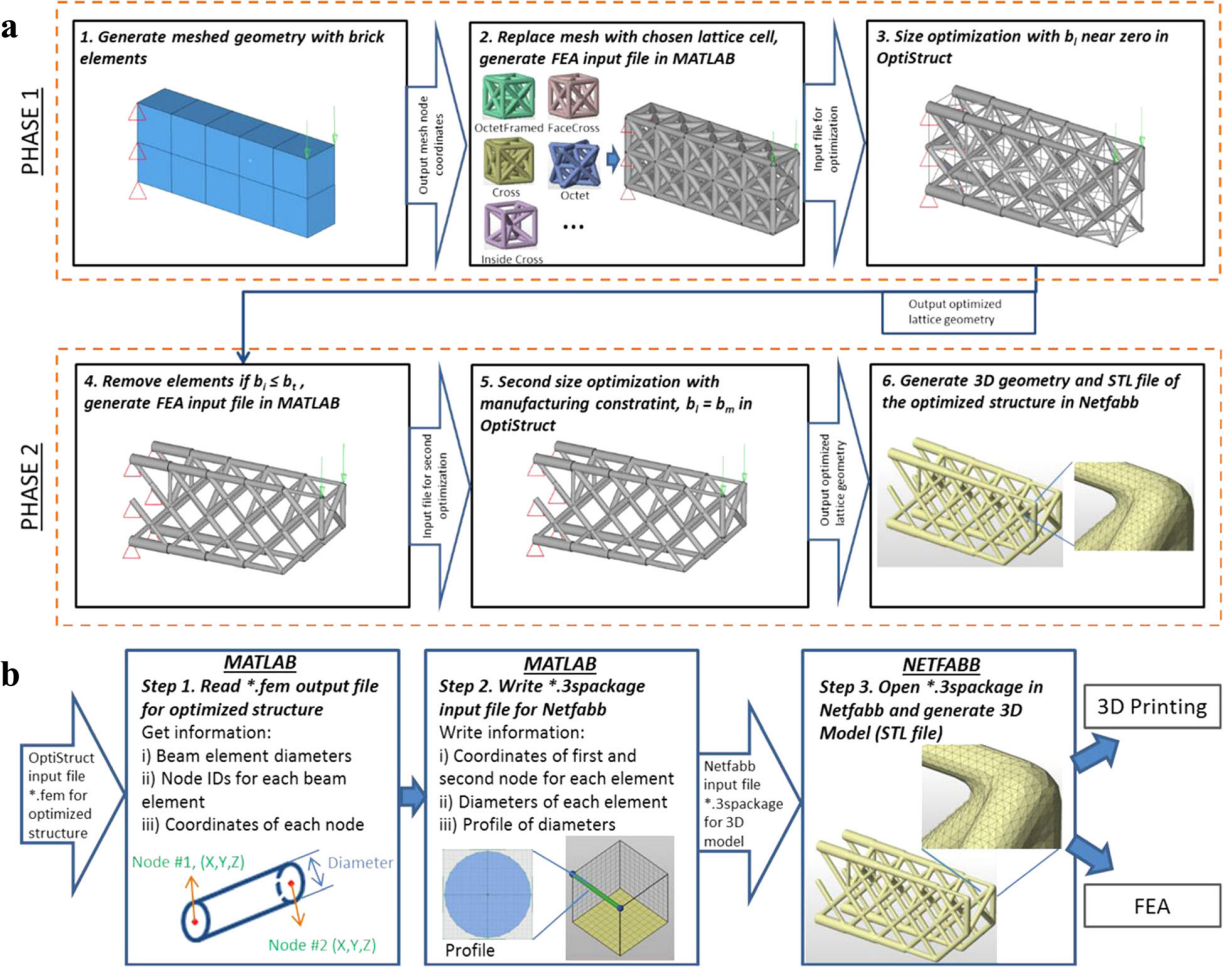
Optimization design method: **a** Lattice design process. **b** Specific steps consist of MATLAB and Netfabb softwares [13]

**Fig. 23:**
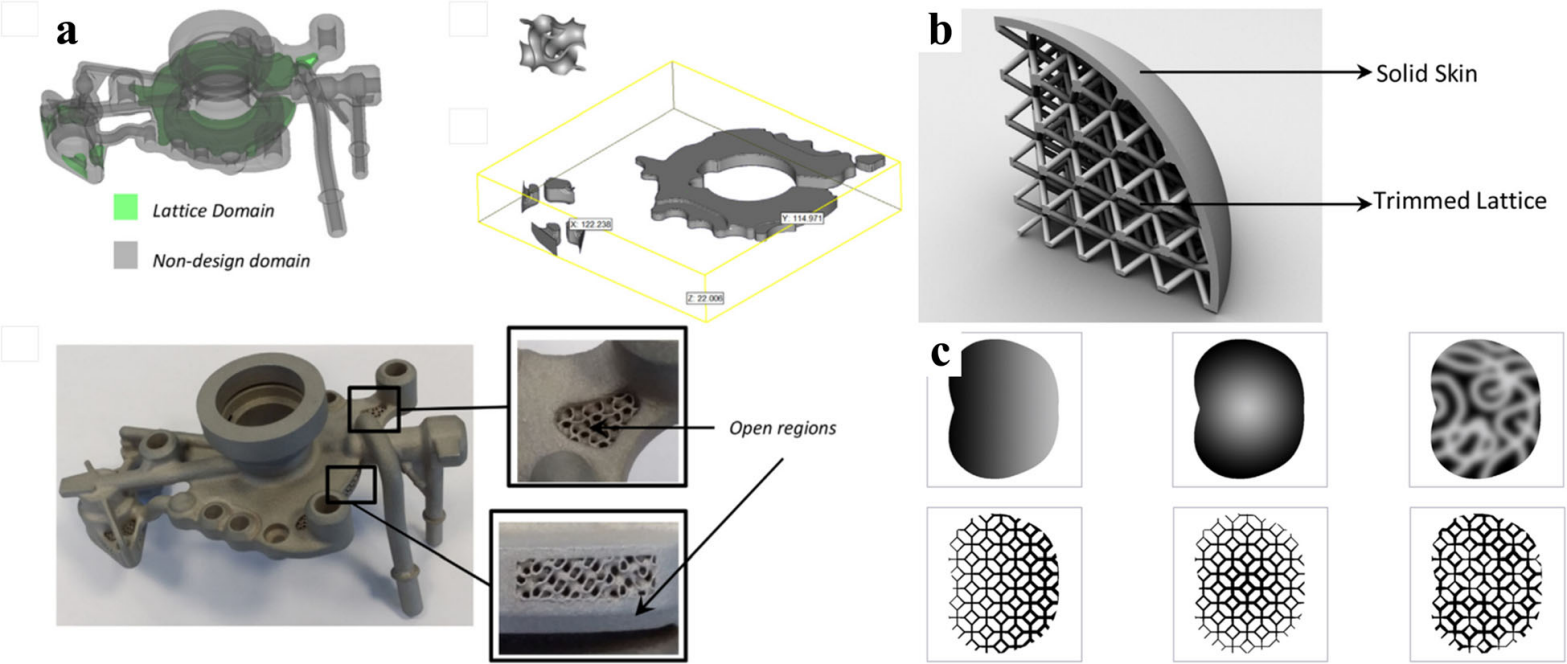
The functionally graded lattice structure design: **a** Product designed with Gyroid lattice. **b** Lattice model with solid skin. **c** Functionally graded structures [52]

**Fig. 24:**
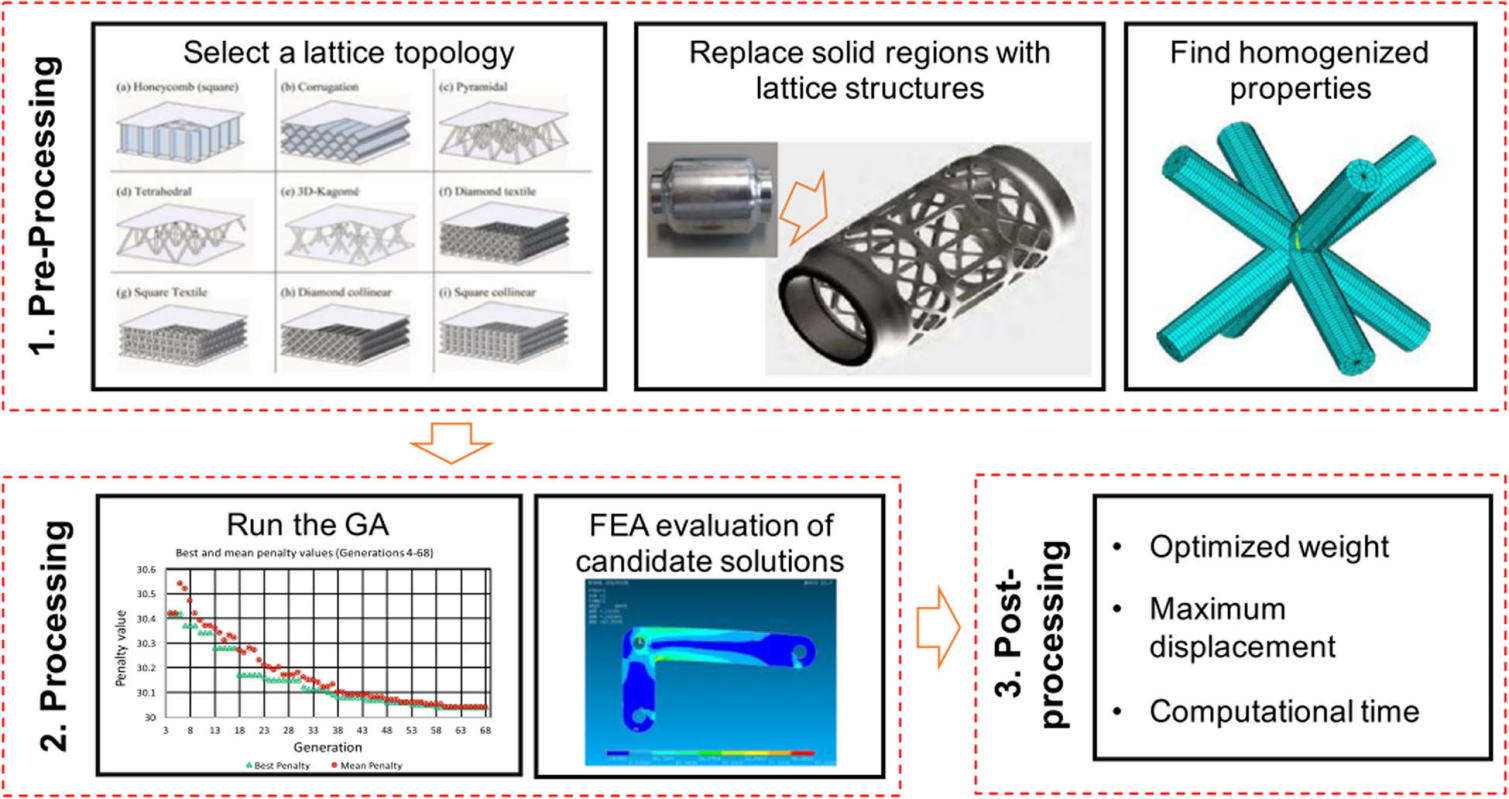
Workflow of the design and analysis of lattice structures [53]

The Voronoi tessellation method is a common method in computer-aided design and graphic domains. Gómez et al. presented a scaffold design method based on the Voronoi algorithm to obtain 3D virtual isotropic scaffolds, which can match the main histomorphometric indices of trabecular bones [36], as illustrated in Fig. 16. The nature bone properties at all levels can be acquired by their designed scaffolds.

### Scaffold design with mechanical analysis

In order to apply porous structures as tissue engineering scaffolds, besides the basic demands of porosity and interconnectivity, the mechanical properties of scaffold should also be taken into consideration. The mechanical properties of the designed scaffold are the significant factors to evaluate the final qualities. Researchers have made numerous attempts to test and optimize the properties of the designed scaffolds [37, 38]. Wang et al. proposed a top-down design method based on Voronoi-Tessellation to generate porous scaffolds [39] with specified and functionally graded porosity, as presented in Fig. 17. The mechanical properties can be controlled by changing the porosity and irregularity. Thus, the compressive properties of the designed porous structures can be adjusted to the level of cortical bone.

For the purpose of designing porous structures as close to the trabecular bone as possible, voxel-wise topology optimization algorithm was utilized to generate sparse yet stable structures [40] as shown in Fig. 18. They designed the internal infill structures with full consideration of the external loads. The generated porous structures are similar not only with the internal pores, but also with the function properties.

Moreover, researchers have also done a lot of work on creating scaffold unit libraries [41, 42], designing heterogeneous porous scaffolds [43–46]. The design of porous scaffold is an interdisciplinary challenge, requiring further research attentions. In order to supply enough space and adequate mechanical support for cell spreading, the deigned intricate scaffolds should better be similar with the actual tissues as close as possible [47].

## Lattice structure design methods

Lattice structures are widely existed in natural structures, with periodical repeated units and complex topology, which are also termed as porous cellular structures [7]. In addition to the applications as lightweight infill and porous scaffolds as mentioned in last two sections, lattice structures can also be adopted to absorb sound [3], dissipate heat, isolate vibration [5], absorb energy [48, 49]. Recently, more and more lattice models are applied as core structures in novel sandwich structures to transverse shear and compression loads for engineering applications [50]. With the rapid development of additive manufacturing, the lattice structure which is difficult to manufacture by typical technology can be efficiently fabricated by 3D printing [1].

Nguyen and Vignat proposed an efficient method to design lattice structures for 3D printing [6]. They summarized the most common applied lattice units as shown in Fig. 19a. For any complex models, the selected unit cells can be designed within the input model after the Boolean operations. According to various mechanical or other physical requirements, diverse unit cells can be adopted or even combined to design the final lattice structures. These lattice units are basically simple bar units, which are too simplistic.

Chen presented a general structure configuration design format to design lattice structures [12], as shown in Fig. 20. Based on the mesh geometric method, conformal lattice structures can be designed efficiently. In order to meet various demands, all of the structure dimension, strut shape, size and connection can be conveniently modified. Different from several existing methods, fillets can be automatically added to optimize the mechanical performances.

The traditional bidirectional evolutionary structural optimization algorithm was utilized and modified to optimize the lattice structures [51], as presented in Fig. 21. The functional roles of the designed solid volume and skin are respectively analyzed in the lattice frame generation method. In particular, the lattice orientation is designed to further improve the lattice performances via changing the strut thickness. The Von-Mises stress can be greatly reduced, while reducing the weight of lattice structures.

Besides the physical requirements, the printability of the technology and equipment is also a crucial issue, which is ignored by above mentioned methods. Gorguluarslan et al. developed a two-phase lattice structure optimization framework with full consideration of the demands of the 3D printing [13], as presented in Fig. 22.

Aremu introduced a novel voxel-based method to design conformal lattice and functionally graded structures [52]. Conventional representation methods for 3D models are not suitable for these intricate lattice structures. With the help of this voxel approach, any type of lattice unit can be efficiently designed with the given model boundary, which is illustrated in Fig. 23a and Fig. 23b. Meanwhile, the functionally graded structures can be designed by changing the size of lattice struts, as shown in Fig. 23c.

Similar with other complex topology structure design problems, the optimization design according to the analysis results is an indispensable process. In most cases, the lattice design is a multipurpose optimization issue, which is very complicated and time-consuming. A user-friendly optimization approach is introduced to solve this problem [53]. In order to reduce the calculation costs, the homogenization properties are analyzed by the finite element method, and the configuration of lattice is optimized by the genetic algorithm, as illustrated in Fig. 24.

Currently, the amount of designed lattice unit type is relatively small, and most of the unit cells are too simple to represent the complex structures exist in the nature. Due to the intricate structures and complex topology, the analysis methods, such as finite element analysis or topology optimization approach are extremely expensive. Most of the current designed lattice structures lack the analyses and optimizations to meet the complicated demands. In addition, current 3D modeling software packages lack the support of lattice structure design. Efficient and convenient design tools or platforms of lattice structures are eager to be developed.

## Conclusion and future work

According to the above brief review discussions, it is found that the complex topology structure design is a significant and interesting topic considering multi-discipline requirements. Although many researchers have dedicated to improve the degree of design freedom and performances of the designed structures, there are still much potential to advance the design methodology. The future work can be implemented according to the following limitation aspects of current approaches.

(1) The final target of designing complex topology structures is to fabricate and then apply them in actual engineering situations. The design method should take full consideration of diverse requirements. More importantly, a balance need to be met when different demands cannot be fully fulfilled at the same time.

(2) Most of current methods are only imitations of existing natural structures, while the performances of the designed structures cannot match that of natural ones. Automated and convenient methods should be developed to generate exactly the same complex topology structures with the natural structures, according to reliable data resources, such as CT or MRI images. This exact design technology is meaningful to repair tissues or bones in human body.

(3) The analysis and optimization design are crucial technologies in complex topology design methodology. Nevertheless, the most common applied finite element method is very time-consuming. And the meshing process has direct influences on the final analysis results. The complex structures further aggravated the burden of the calculations. A lightweight and convenient analysis platform should be developed to efficiently acquire the calculation results. Many researchers have attempted to analyze the performances with novel methods, such as topology optimization and cross-section methods. And the recent artificial intelligence or machine learning methods may be utilized to achieve the analysis and optimize task in the future.

(4) With respect to the data structures of complex topology structures, current mesh models or parametric models are not ideal. More efficient and convenient data format should be developed to accurately describe the designed structures with small amount of data. Meanwhile, the data structure should be compatible to the current system and equipment of 3D printing.
